# Effects of Vitamin C on the Prevention of Ischemia-Reperfusion Brain Injury: Experimental Study in Rats

**DOI:** 10.1155/2019/4090549

**Published:** 2019-12-15

**Authors:** Kelston Paulo Felice de Sales, Bruno Araújo Serra Pinto, Nathalee Liberal Xavier Ribeiro, Thamys Marinho Melo, Leonardo Victor Galvão-Moreira, Sebastião Barreto de Brito Filho, Flávio Nigri

**Affiliations:** ^1^Department of Vascular and Endovascular Surgery, University Hospital, Federal University of Maranhão, São Luís, Brazil; ^2^Laboratory of Experimental Physiology, Department of Physiological Sciences, Federal University of Maranhão, São Luís, Brazil; ^3^School of Medicine, Federal University of Maranhão, São Luís, Brazil; ^4^Department of Medicine II, Federal University of Maranhão, São Luís, Brazil; ^5^Head of Neurosurgery Teaching and Assistance Unit, Department of Surgical Specialties, Pedro Ernesto University Hospital, Nervous System Electric Stimulation Laboratory (LabEEL) – Neurosurgery Teaching and Assistance Unit, Pedro Ernesto University Hospital, Rio de Janeiro State University, Rio de Janeiro, Brazil

## Abstract

**Background:**

Reperfusion syndrome after carotid endarterectomy is a complication associated with cerebrovascular self-regulation in a chronically hypoperfused cerebral hemisphere, leading to severe neurological damage. Vitamin C is an important antioxidant in brain metabolism that has shown some neuroprotective actions.

**Objective:**

To investigate the potential effects of vitamin C on cerebral reperfusion in comparison with placebo (saline) in rats.

**Methods:**

Male Wistar rats were divided into 3 groups: (i) Sham (*n* = 4), animals exposed to carotid arteries dissection without clamping; (ii) Control (*n* = 4), animals exposed to carotid arteries dissection without clamping; (ii) Control (*n* = 4), animals exposed to carotid arteries dissection without clamping; (ii) Control (

**Results:**

Rats treated with vitamin C presented with a similar behavior as compared to the Sham group in all the three tests (*p* > 0.05), but it was significantly different from controls (*p* > 0.05), but it was significantly different from controls (*p* > 0.05), but it was significantly different from controls (

**Conclusion:**

In the present study, vitamin C was associated with behavioral and motor preservation as well as decreased cerebral MDA levels after induced cerebral ischemia in rats.

## 1. Introduction

Carotid atherosclerotic disease is a leading cause of stroke, accounting for 20−30% of all cases [1, 2]. Notably, patients submitted to carotid endarterectomy have shown reduced rates of stroke and death, similarly to those who underwent stenting [3]. Nonetheless, temporary artery occlusion can trigger lesions resulting from cerebral ischemia followed by reperfusion, characterizing a postoperative cerebral hyperperfusion syndrome (CHS). Indeed, individuals who undergo carotid revascularization with endarterectomy are more likely to develop CHS as compared to those treated with carotid stent angioplasty [1, 4].

CHS following carotid endarterectomy occurs more frequently in chronic hypertensive patients and/or in those with greater carotid stenosis, being characterized by severe ipsilateral headache, seizures, and intracranial hemorrhage [5–7]. Increased nitric oxide levels during clamping of internal carotid artery and release of oxygen-derived free radicals produced during brain perfusion restoration are involved in endothelial dysfunction and deterioration of post-endarterectomy autoregulation mechanisms [8]. Higher production of reactive oxygen species (ROS) and proinflammatory cytokines are some of the major regulating mechanisms of inflammation induced by ischemia-reperfusion [9]. Still, although CHS is relatively uncommon, it may be associated with severe outcomes, especially in asymptomatic carotid stenosis [7, 10].

Nevertheless, preventive protocols for CHS remain not well defined. Exogenous antioxidants such as ascorbic acid (vitamin C), *α*-tocopherol (vitamin E), *β*-carotene, resveratrol, and canabidiol may prevent ROS-induced neuronal cell damage and rapid consumption of endogenous antioxidant enzymes [11–13]. Vitamin C is an important antioxidant in brain metabolism, whose neuroprotective actions on reperfusion syndrome have been demonstrated [14, 15]. Intraperitoneal injection of vitamin C was effective when compared to either intravenous or oral administration, reaching a maximum concentration 60 min after administration [16, 17].

ROS can be either a cause or a consequence of conditions associated with oxidative stress such as cerebral ischemia. Among the most widely used methods for indirect measurement of ROS and membrane lipoperoxidation are the spectrophotometric and chromatometric techniques that measure tissues and/or biofluids concentrations of tripeptides such as malondialdehyde (MDA) [18, 19]. Therefore, the present study was aimed at investigating the neuroprotective effect of vitamin C for prevention of brain damage after ischemia induced in rats by evaluating motor/behavioral changes and MDA levels.

## 2. Methods

For this experiment, carried out from June to August 2018, 6-month-old male Wistar rats with approximately 485 g were used (*n* = 12). Animals were obtained from the local animal house at the Federal University of Maranhão, São Luís, Brazil, and maintained at the post-graduation laboratory in polyethylene cages lined with xylan and environmental enrichment (4 animals per cage) in a controlled environment (21−23°C, 12h light/dark cycle) with free access to water and food. All experimental protocols involving animal handling were previously approved by the local Ethics Committee on Animal Use (CEUA), process #23115.001635/2016-16.

### 2.1. Drugs and Reagents

The following chemical products were used: salicylate 0.9%, vitamin C 100 mg/mL (Farmace, Barbalha, CE, Brazil), isoflurane 100% 1 ml/mL (Cristália, Itapira, SP, Brazil), ketamine (Ceva, Paulínia SP, Brazil), xylazine 1% (König, Marinque, SP, Brazil), and chlorhexidine digluconate 10 mg/mL (Neo Química, Anapolis, GO, Brazil). The hippocampus and striatum were homogenized in 20 mM phosphate buffer solution (PBS) containing 140 mM potassium chloride (pH 7.4, 1 : 10 weight/volume). In the analysis of MDA levels, solutions of 0.01% butylated hydroxytoluene, 1 mM EDTA, 10% trichloroacetic acid, 0.67% thiobarbituric acid, 7.1% sodium sulfate, butanol PA, and 1, 1, 3, 3 tetramethoxypropane (Sigma-Aldrich, St. Louis, MO, USA) were utilized.

### 2.2. Experimental Design and Treatments

Animals were randomly assigned into 3 groups of 4 animals each and then anesthetized with isoflurane 1.3−1.5% in 100% oxygen and 0.9% saline solution. Next, the Sham group received an intraperitoneal injection of 0.9% saline solution (0.1 mL/kg) 30 min prior to bilateral carotid arteries dissection without clamping. Control group also received an intraperitoneal injection of saline (0.1 mL/kg) and underwent bilateral dissection of common carotid arteries by clamping with clips for 20 min followed by further surgical exposure and removal of clips. Vitamin C group received an intraperitoneal injection of vitamin C (750 mg/kg) 30 min before bilateral dissection of common carotid arteries by clamping with clips for 20 min followed by surgical exposure and removal of clips. During application and removal of clips, animals were kept in individual cages under heating and reduction of luminosity.

### 2.3. Anesthetic and Surgical Procedures

Hypotensive anesthesia was induced in rats by inhalation of a mixture of isoflurane and oxygen using a Fuji anesthesia apparatus (Takaoka, São Paulo, SP, Brazil) connected to a plastic mask adapted to the nose of each animal. Next, tricotomy was performed in the cervical region followed by antisepsis with alcoholic cloroxidine and median cervix incision with dissection until isolation of common carotid arteries. In the Sham group, clamping of carotid arteries was not performed. In the other groups, transient global cerebral ischemia was induced through bilateral artery occlusion by applying a clip in each common carotid artery.

A vaporizer was set to release a minimum airflow (2.0 L/min). The mixture delivered to each animal was monitored to maintain the minimum concentration of isoflurane required for an efficient anesthesia (assessed by squeezing the animal's tail). Under such conditions, animals were fixed in a stereotactic structure and anesthetized with 1.3−1.5% isoflurane in 100% oxygen and 0.9% saline for the time required in order to make a ventral neck incision for exposing the common carotid arteries [12]. The temperature in the surgical field was maintained at 37.5°C during surgery by light heating.

Cerebral ischemia was induced for 20 min through bilateral common carotid artery occlusion using aneurysm clips (ADCA, Belo Horizonte, MG, Brazil). During the occlusion procedure, rats were kept in a heating box (internal temperature 30 ± 1°C) to prevent cerebral hypothermia induced by ischemia [20]. After 20 min of clamping, rats were anesthetized again and the previous incision was reopened. Clips were removed and carotid arteries were visually inspected for reperfusion, while the incision was closed using sutures [21]. The animals were then placed back in cages.

### 2.4. Behavioral Assessment

One week prior to surgical procedures, the animals underwent appropriate training for behavioral assessment. After 24 hours of surgical procedures, they were submitted to behavioral evaluation and then killed by decapitation under deep anesthesia.

#### 2.4.1. Open Field Test

This test has been utilized to estimate locomotor activity, willingness to explore, and anxiety-like behavior in rodents by measuring the behavior of an animal after it enters an open and novel arena [22]. Animals were placed for 5 min at the center of an open field circular arena, divided in 9 quadrants. Next, the total number of quadrant crossings, frequency of entrances in the central quadrant and stereotyped behaviors were recorded [23].

#### 2.4.2. Morris Water Maze Test

The water maze test has been widely used for assessing memory, learning, and different forms of cognition. It is aimed at determining the learning memory for rats to locate a hidden platform in a water tank using distal cues that are placed around the water maze after repeated trials over a certain period of time [24, 25]. The experiment was conducted as previously described [23, 26]. Briefly, a hidden escape platform was submerged in a pre-set quadrant of a circular water pool divided into 4 quadrants. Animals were released into the water from one of the 4 quadrants, facing maze wall, and allowed to swim for 120 s to find the hidden platform (4 trials/day for 3 days). Those who failed were guided to the platform and allowed to stay on it for 30 s. Latency to find the hidden platform was recorded for spatial learning assessment. At day 4, the platform was removed and the animals were released to swim for 120 s. For memory retention assessment, the time spent in the quadrant where the platform was expected to be and the number of entries in the target quadrant were obtained.

#### 2.4.3. Rotarod Test

The rotarod test is a highly sensitive method to evaluate strength, coordination, and balance, being useful for determining vestibulomotor function in rats following posttraumatic brain injury by detecting deficits when other tests show normal vestibulomotor function [25]. For this experiment, rats were pretrained on a rotarod (12 rpm, 5 min) 2 days prior to surgical procedures, and, after the intervention, 3 times a day for 5 days. At day 5, animals were fasted for 2 h before the experiment and latency to fall was recorded in seconds [23].

### 2.5. Euthanasia, Isolation of Brain Areas and Preparation of the Homogenate

After behavioral tests, animals were craniotomized under deep anesthesia by ketamine (120−160 mg/kg) and xylazine (30−40 mg/kg) overdose. Rats then had their brain removed from the skull and both the hippocampus and striatum was isolated, washed with saline, homogenized in 20 mM PBS containing 140 mM potassium chloride, and frozen for further utilization.

### 2.6. Analysis of MDA Levels in Cerebral Tissues

MDA levels in hippocampus and striatum were measured as previously described [27]. Homogenized tissues were centrifuged, then 0.01 vol% butylated hydroxytoluene and 1 mM EDTA were added to the buffer. The supernatant (150 *μ*L) was added to cold 10% trichloroacetic (300 *μ*L) acid and 0.67% thiobarbituric acid in 7.1% sodium sulfate and then incubated, cooled, and the pink chromogen was isolated by the addiction of buthanol and centrifugation. TBARS levels were analyzed using a fluorimetric assay (*λ*ex 515 nm, *λ*em 553 nm). The calibration curve was determined by 1, 1, 3, 3-tetramethoxypropane as standard and TBARS levels were expressed in *μ*M MDA.

### 2.7. Statistical Analysis

Statistical analysis was conducted using the GraphPad Prism 7.0 software (GraphPad Software Inc., San Diego, CA, USA). The Kolmogorov-Smirnov was used to test the normality of variables, and was followed by one-way ANOVA and the Newman–Keuls post-hoc test at a 5% significance level.

## 3. Results

**Figure 1:**
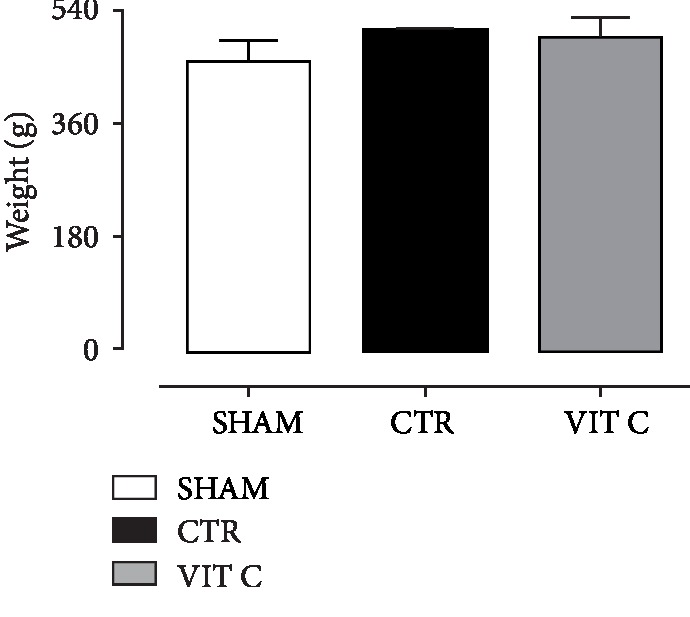
Morphometric parameters. A, body weight (BW, g) assessed in 3 groups of 4 animals each: SHAM, Control (CTR), and Vitamin C (VIT C). Bars represent mean ± SEM (one-way ANOVA – Newman Keuls). ^∗^*p* < 0.05 when compared to SHAM; ^#^*p* < 0.05 when compared to CTR.

**Figure 2:**
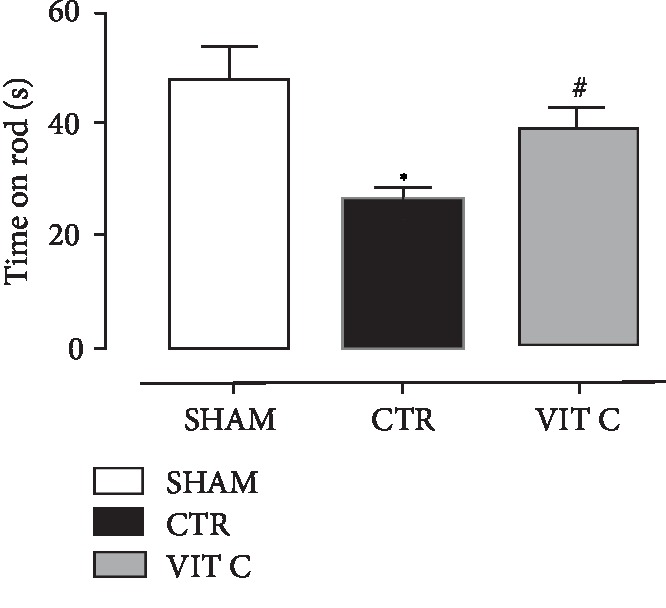
Motor assessment. Motor skills and equilibrium assessment by time on rod (s) in rotarod test assessed in 3 groups of 4 animals each: SHAM, Control (CTR), and Vitamin C (VIT C). Bars represent mean ± SEM (one-way ANOVA – Newman Keuls). ^∗^*p* < 0.05 when compared to SHAM; ^#^*p* < 0.05 when compared to CTR.

**Figure 3:**
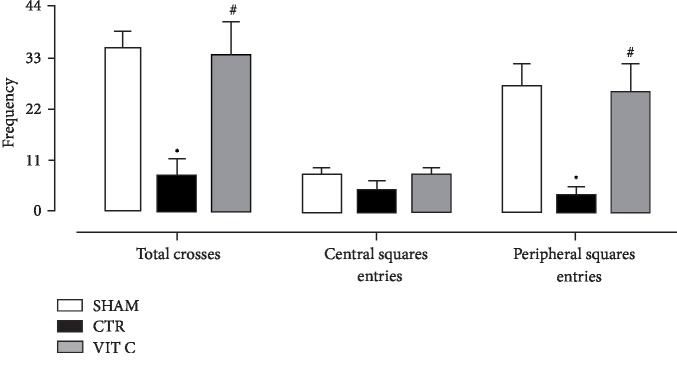
Exploratory behavioral assessment. Exploratory behavior assessed by frequency of total crosses, central and peripheral squares entries in the open field test assessed in 3 groups of 4 animals each: SHAM, Control (CTR), and Vitamin C (VIT C). Bars represent mean ± SEM (one-way ANOVA – Newman Keuls). ^∗^*p* < 0.05 when compared to SHAM; ^#^*p* < 0.05 when compared to CTR.

As displayed in Figure 1, no statistical significant difference was found in rats' body weight between the groups (*p* > 0.05), suggesting that the surgical procedure and treatments did not affect weight gain of animals.  Figure 2 illustrates the comparison of the rotarod test scores. Vitamin C group presented with a similar motor behavior as compared to the Sham group (*p* > 0.05), whereas the Control group showed a more compromised motor behavior (*p* < 0.05).  Figure 3 shows the results for the open field test. It was observed that both the number of total crosses and peripheral square entries of rats treated with vitamin C were similar to those from sham-operated rats (*p* > 0.05), but statistically different from the controls (*p* < 0.05). The number of central square entries was similar among all groups (*p* > 0.05).

**Figure 4:**
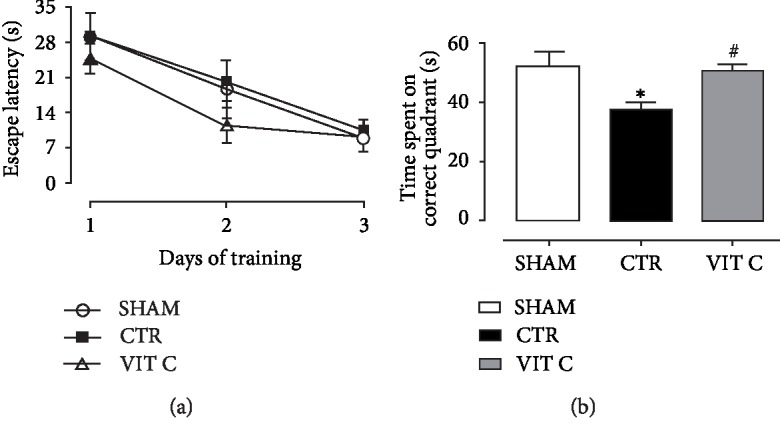
Cognitive function assessment. (a) Spatial learning assessed by latency time (s) to find the hidden platform in water maze test; and (b) memory assessed by time spent (s) in the correct quadrant in water maze test in 3 groups of 4 animals each: SHAM, Control (CTR), and Vitamin C (VIT C). Points and bars represent mean ± SEM (one-way ANOVA – Newman Keuls). ^∗^*p* < 0.05 when compared to SHAM; ^#^*p* < 0.05 when compared to CTR.

**Figure 5:**
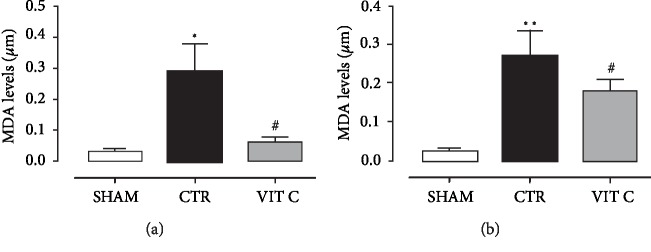
Redox profile. Lipide peroxidation assessment by fluorimetric quantification of Malondialdehyde (MDA, *μ*m) in homogenated samples of hippocampus (a) and striatum (b) in 3 groups of 4 animals each: SHAM, Control (CTR) and Vitamin C (VIT C). Points and bars represent mean ± SEM (one-way ANOVA – Newman Keuls). ^∗^*p* < 0.05 when compared to SHAM; ^∗∗^*p* < 0.01 when compared to SHAM; ^#^*p* < 0.05 when compared to CTR.

Results for the Morris water maze test are illustrated in Figure 4. After 3 days of training, escape latency time to find the hidden platform was reduced in all groups. However, time spent in the correct quadrant was lower in the Control as compared to the other groups (*p* < 0.05), but it was similar between the Sham and Vitamin C groups (*p* > 0.05).  Figure 5 shows that vitamin C reduced MDA levels in both the hippocampus (A) and striatum (B) in comparison with placebo (*p* < 0.05). MDA levels in the group treated with vitamin C were similar to the Sham group (*p* > 0.05).

## 4. Discussion

CHS is a complication that can occur after carotid endarterectomy, which has been associated with ROS release [8, 9]. In the present investigation, we evaluated whether the treatment with vitamin C could preserve the motor/behavioral parameters and modulate MDA levels in rats that underwent carotid arteries dissection with temporary clamping. Collectively, our findings show that rats exposed to carotid arteries dissection with clamping and treated with vitamin C presented with preserved motor and behavioral parameters as well as decreased MDA levels when compared to rats treated with placebo.

Natural products have been widely screened for antioxidant properties, and several studies have tested them as potential pharmacological approaches for preventing ischemia-reperfusion brain injury [28]. For example, fish oil reversed oxidative damage to control levels, attenuated oxidative stress after 24 h of reperfusion and prevented retrograde amnesia several weeks later, demonstrating its long-term positive effects on the hyperacute phase of cerebral ischemia-reperfusion [29]. Mangiferin, a natural glucosyl xanthone, reduced MDA content and pro-inflammatory cytokines in brain tissues of rats with cerebral ischemia-reperfusion injury, indicating that it might play a protective effect on CHS [9].

Neuroprotection by vitamin C has been recently demonstrated *in vitro* and *in vivo*. Vitamin C inhibited mitochondrial damage and cell death against oxidative injury *in vitro* [30]. Regarding the potential action of vitamin C on CHS, a previous study investigated the effects of ascorbic acid niosomes on cerebral ischemia-reperfusion in male rats and found that niosomes had more neuroprotective effects against this lesion than free ascorbic acid [13]. However, this study included only electron micrograph and histologic analysis of neurons, while either motor or behavioral parameters were not evaluated.

Additionally, vitamin C has been linked to the reduction of ischemia-induced edema, the protection of cell death against neurotoxicity following ischemia, and the improvement of neuron's synaptic connection in cerebral ischemia [14]. In the current study, vitamin C was capable to reduce oxidative stress, which might partially explain its neuroprotective effects on the CHS model developed. Importantly, vitamin C reduced oxidative stress, improved vascular function and structure, and prevented progression of hypertension in stroke-prone spontaneously hypertensive rats, effects of which were mediated through modulation of ROS release [31].

Moreover, in diabetic rats, daily intake of ascorbic acid reduced the severity of cerebral ischemic injury, and this effect was related to anti-apoptotic and anti-inflammatory actions as well as the reduction of oxidative stress in the brain [32]. Treatment with vitamin C and an adenosine receptor agonist attenuated ischemia-induced brain injury through antiapoptotic actions and improved memory loss, thereby showing a potential neuroprotective effect [33].

In order to evaluate the effects of transient global cerebral ischemia, it is important to conduct behavioral tests. The effects of bilateral common carotid artery occlusion were transient in spatial memory and anxiety-like behavior remained elevated 28 days after reperfusion, which may be related to hippocampal injury in mice. It induced cognitive damage in passive avoidance and spatial tasks, and increased the latency to find the platform and time spent in the correct quadrant in the water maze test, indicating learning and memory deficits, respectively [34]. In this study, bilateral common carotid artery occlusion followed by reperfusion promoted significant impairment of sensorimotor activities, corroborating with earlier reports showing neurological deficits following ischemia-reperfusion brain injury [35].

Furthermore, few studies have investigated neuronal damage outside the hippocampus in relation to behavioral tasks after global ischemia or demonstrated changes induced by ischemia of cellular activity in brain regions outside the hippocampus, which could have a significant impact on behavioral recovery [34]. Impressively, ascorbic acid injection significantly decreased the number of both necrotic and apoptotic cells in cortex, caudate putamen, thalamus, and hippocampus in newborn rats with cerebral hypoxic-ischemia [36].

It is worth mentioning that this study has several limitations, including the reduced number of animals per group and the fact that other biomarkers for oxidative stress were not evaluated. MDA is the most frequently used biomarker of oxidative stress in several diseases; however, its sensibility is highly controversial due to high reactivity and possibility of various cross-reactions [37].

On the other hand, a reproducible model of transient global cerebral ischemia was used, which is essential for elucidating the molecular mechanisms of ischemic brain injury and for discovering new treatments. The rat model of bilateral common carotid artery occlusion combined with isoflurane-induced hypotension leads to hippocampal damage in adult rats, and thereby can be used for pharmacological screening [35]. In addition, prior reports tested lower doses of vitamin C as compared to this study, which showed a marked neuroprotective effect of a high dose of vitamin C in a rat model of CHS.

## 5. Conclusion

In summary, the administration of vitamin C promoted a neuroprotective action against ischemia-reperfusion brain injury in adult rats as assessed by behavioral changes associated with mobility and variations obtained in enzymatic dosages, especially those related to the hippocampus. Further studies are thus necessary to confirm the potential benefic effects of vitamin C on CHS.

## Data Availability

Data used to support the findings of this study are available from the corresponding author upon request.

## Additional Points

The current study did not include any experiment with humans or samples obtained from humans. All experiments carried out with animals were in accordance with the US National Research Council's “Guide for the Care and Use of Laboratory Animals”.
